# A comprehensive review on recent advances in exosome isolation and characterization: Toward clinical applications

**DOI:** 10.1016/j.tranon.2024.102121

**Published:** 2024-09-14

**Authors:** Nihat Dilsiz

**Affiliations:** Experimental Medicine Application and Research Center (EMARC) Validebag Research Park, University of Health Sciences, Istanbul, Turkey

**Keywords:** Liquid biopsy, Extracellular vesicles, Exosomes, Isolation, Characterization, Biomarkers, Cancer

## Abstract

Exosomes are small, round vesicles in the 30 and 120 nm diameter range released by all living cell types. Exosomes play many essential functions in intercellular communication and tissue crosstalk in the human body. They can potentially be used as strong biomarkers and therapeutic agents for early diagnosis, therapy response, and prognosis of different diseases. The main requirements for exosomal large-scale clinical practice application are rapid, easy, high-yield, high purity, characterization, safety, low cost, and therapeutic efficacy. Depending on the sample types, environmental insults, and exosome quantity, exosomes can be isolated from various sources, including body fluids, solid tissues, and cell culture medium using different procedures. This study comprehensively analyzed the current research progress in exosome isolation and characterization strategies along with their advantages and disadvantages. The provided information will make it easier to select exosome separation methods based on the types of biological samples available, and it will facilitate the use of exosomes in translational and clinical research, particularly in cancer.

**Lay abstract** Exosomes have recently received much attention due to their potential to function as biomarkers and novel therapeutic agents for early diagnosis, therapeutic response, and prognosis in various diseases. This review summarizes many approaches for isolating and characterizing exosomes, focusing on developing technologies, and provides an in-depth comparison and analysis of each method, including its principles, advantages, and limitations.

## Highlights

• Exosomes play many essential functions in intercellular communication and tissue crosstalk in the human body.
• They can potentially be used as strong biomarkers and therapeutic agents for early diagnosis, therapy response, and prognosis of various diseases.
• Exosome isolation is likely to demand more exact and extensive procedures for use in therapy than those needed for diagnosis.
• This review covers recent conventional methods that have been employed by laboratories worldwide to separate, detect, and characterize exosomes.

## Graphical abstract

**Figure:**
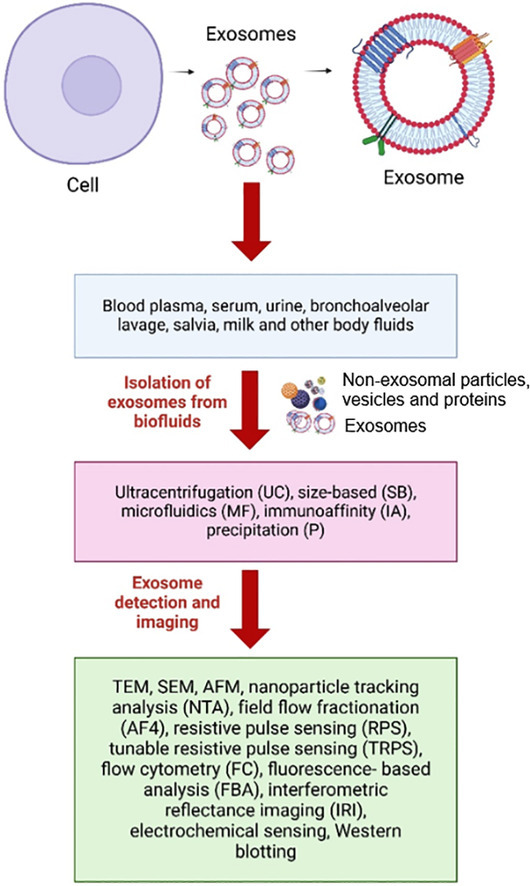

## Introduction

Extracellular vesicles (EVs) are typically classified into three distinct groups: exosomes (∼30–120 nm), microvesicles (∼100–1000 nm), and apoptotic bodies (∼500–3000 nm). These three main subtypes are differentiated by their biogenesis, release pathways, size, morphology, surface biomarkers, content, and function. Each cell produces and releases exosomes into biological fluids, including blood, saliva, breast milk, urine, lymph, vitreous, bile, and cerebrospinal fluid. According to Kumar et al., the concentration of exosomes in the serum of healthy human blood samples was reported to be as high as 7 × 10^8^ particles/ml [1]. Exosomes are formed by late endosomes' inward budding, leading to intraluminal vesicles (ILVs) forming in the early multivesicular body (MVB) membrane. These exosomes are then released upon fusing the MVB's limiting membrane with the cellular membrane. In contrast to exosomes, microvesicles form directly from the cellular membrane and enter the extracellular space [2]. Despite their different subcellular origin pathways, exosomes and microvesicles may share similarities in size, content, and molecular regulation, making it difficult to separate them after release.

Exosomes can be transferred from the host to destination cells, which results in the transfer of epigenetic information and the reprogramming of cellular activities of the recipient cells [3]. Exosomes transport various cargo molecules, including metabolites, DNA fragments, coding messenger RNAs (mRNAs), noncoding long RNAs (lncRNAs) and microRNAs (miRNAs), proteins, and lipids, from their originating cells to target cells [[4], [5], [6], [7]]. Exosomes have recently gained much attention as mediators of intercellular communication in physiologically healthy conditions and under pathophysiological stress [[8], [9], [10]]. They play a critical role in many processes that contribute to cancer progression, including the maintenance of cancer metastasis, the emergence of treatment resistance, inflammatory response, and immunological regulation [3,11,12]. Exosomes are now being used in more than 400 clinical trials for various disorders, either as direct therapeutic mediators or as biomarkers for therapy response (www.globaldata.com; www.clinicaltrials.gov).

**Fig. 1:**
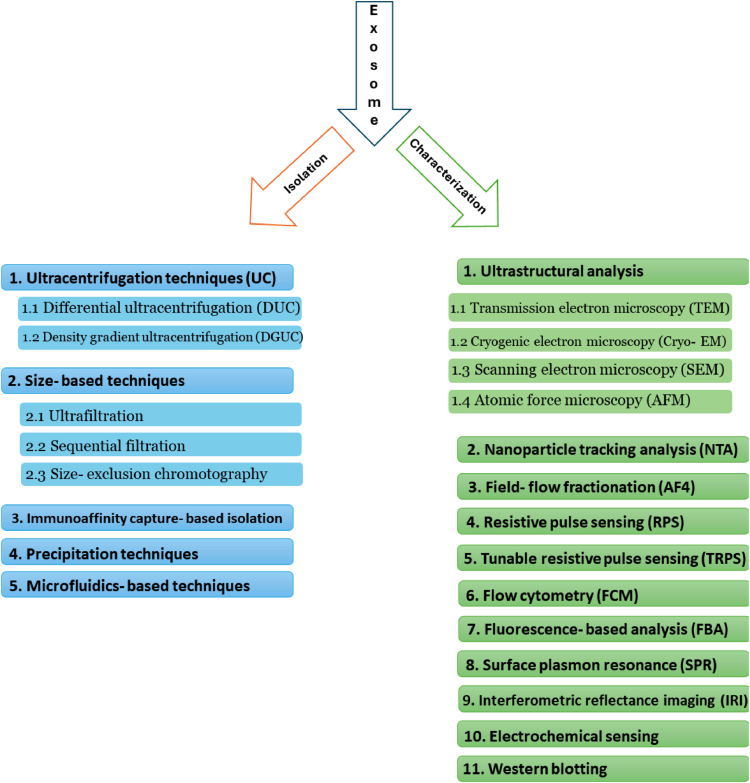
Isolation and characterization methods for exosomes.

Exosomes must be separated from non-exosomal components, such as microvesicles, apoptotic bodies, and other biomolecules in sufficient quantities, purity, and size to perform fundamental research [13]. The ideal standardized isolation method for exosome research should be easy and rapid, with high throughput, purity, recovery rates, and low procedure cost. Ultracentrifugation (UC), ultrafiltering (UF), immunoaffinity capture (IA), size-exclusion chromatography (SEC), polyethylene glycol (PEG), microfluidics‑based techniques (MF), and microchip-based (MC) methods are some of the approaches for exosome isolation that have been established (Fig. 1). Each method has its isolation principles with advantages and limitations [14]. Exosome isolation is also possible with various commercial kits [14]. Williams and coworkers examined various exosome isolation methods (UC, UF, SEC, PEG, and AI) in terms of yield and purity of tetraspanin biomarkers (CD9, CD63, and CD81) as measured by Western blotting [15]. They discovered that SEC had the highest yield of these biomarker proteins compared to all other isolation methods.

Because of their nanoscale size, extensive heterogeneous properties, and limited availability in bodily fluid samples, the effective and reliable isolation of pure exosomes continues to be challenging [16,17]. Therefore, more research needs to be done on the exosome isolation technique. This review aims to offer advice on these important methodological concerns and to highlight crucial factors to consider when designing experiments for exosome isolation and characterization (Fig. 1).

## Isolation of exosomes from biological materials

The best strategy for separating exosomes should be chosen depending on which bodily fluid or tissue is used as a source. This section will discuss techniques commonly employed in isolating exosomes from cell culture supernatants, bodily fluids (blood, urine, cerebrospinal fluid, saliva, synovial fluid, milk), or solid tissue samples.

### Isolation of exosomes from the cell line models

Several methods have been developed for isolating exosomes from cell culture models (eukaryotic and prokaryotic cells), each with advantages and limitations. Although many additional extracellular particles may have similar properties to exosomes, most of these approaches are focused on separation by their size and density. In the first step, cells and their debris can be removed from cell cultures by centrifuging them at 300x g and then at 2,000x g for 10 min at 4 °C after 24–48 h incubation. After that, the supernatant, including exosomes is transferred to a new tube and processed through the preferred exosome separation method or stored at -20 °C until it is needed again. Although *in vivo* physiology differs from the cell culture environment, using cell culture media as a source of EVs enables more tightly controlled EV production circumstances. Due to the difficulty in removing contaminating serum exosomes from other EVs, proteins, and lipoproteins, it is recommended to extract exosomes from cells grown in a chemically defined medium when highly pure exosomes are needed for omics analysis or functional investigation. Therefore, it is necessary to use procedural regulations to examine for any possible contamination [18,19].

### Isolation of exosomes from blood

Blood is the most extensively studied biological material in EV research. Preanalytical methods are a crucial factor to consider when separating EVs from blood [20,21]. For example, EVs can be extracted from both serum and plasma. Still, serum preparation induces platelet aggregation, which forms significant amounts of EVs produced from platelets, and the thrombosis that is created traps part of the EVs [7,22,23]. Concerning the exosome isolation studies in human blood samples, 34% have been carried out using blood serum and 62% in blood plasma [24]. The process that causes blood and plasma to clot is known as coagulation. Fibrinogen, a common soluble protein present in plasma, converts into an insoluble fibrin polymer mesh without anticoagulation. The type of anticoagulant (e.g., coumarins, indandiones, and heparins) used can impact the quantity of EVs extracted from plasma, and extreme caution should be avoided due to the risk of hemolysis and platelet activation [25].

**Fig. 2:**
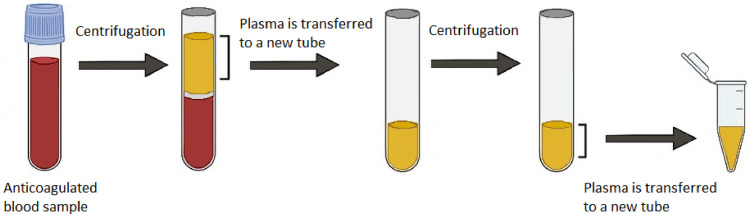
Plasma separation from the blood sample.

In brief, 4–5 ml of blood can be collected from overnight fasted (to reduce the amount of lipoproteins) donors into vacuum polypropylene tubes containing ethylenediaminetetraacetic acid (EDTA) via venipuncture using a 21 G butterfly needle to avoid platelet activation and hemolysis. During blood collection and processing, red blood cells may hemolyze, releasing internal substances like hemoglobin causing serum or plasma to appear reddish rather than yellow. On the other hand, platelets are sensitive to cold activation; therefore, blood tubes should not be stored on ice or in the refrigerator, and all processes should be carried out at room temperature. The plasma is then separated from the blood cells, leukocytes and erythrocytes (which account for approximately 45% of the blood volume), and platelets (the smallest type of cells or cell fragments; 1–3 μm) by spinning the blood in EDTA tubes for 15 min at 4 °C at 1,200x g [25,26]. The separated plasma sample fraction, including the Protease/Phosphatase Inhibitor Cocktail, is centrifuged at 2000 g for 10 min. Then, the supernatant is spun through a 0.45-μm Corning Costar Spin-X centrifuge tube filter (Sigma) at 2,000x g for 10 min to discard cells or cell debris. The separation of EVs is significantly hampered by the presence of substantial amounts of lipoproteins and other proteins and chemicals in plasma, as it contains only 10^8^–10^10^ small EVs/mL and 10^6^ large EVs/mL compared to 10^16^ lipoprotein particles/mL [23,[27], [28], [29]]. Finally, the clear supernatant (plasma) is aliquoted and transferred into the new tubes, snap-frozen in liquid nitrogen, and then stored at -80 °C until further analysis [[30], [31], [32]] (Fig. 2).

### EV isolation from tissue

Understanding both local and distant roles EVs play in forming cancer, neurodegenerative disorders, and cardiovascular diseases has generated significant research interest in separating EVs from tissues. It is important first to validate their presence *in situ*. For instance, electron microscopy can reveal the presence of vesicle formations in pathologic samples including cancer, atherosclerotic plaques, ischemic heart, and brain or muscle tissue [33,34]. It is possible to release EVs through gentle mechanical tissue rupture that an enzymatic process may follow [35]. Isolating EVs directly from fresh tissues is significantly more difficult since tissue is a highly complex structure and EVs must be released from the extracellular matrix rather than tissue homogenization.

Approximately 0.5–1.0 g of the frozen or fresh tissue samples are cut into small pieces (∼ 2 mm sections) using tissue homogenizers and incubated for 30 min in the incubation shaker at 37 °C with protease (e.g., collagenase) and phosphatase inhibitors. The samples are then centrifuged for 10 min at 500x g to precipitate the cells. The collected supernatant of each sample is centrifuged again at 3,000x g for 20 min to precipitate large structures such as apoptotic vesicles. The supernatants are centrifuged at 10,000x g for 40 min to precipitate structures larger than exosomes. The supernatants are filtered with a 0.8 µm filter (Millipore) and exosomes can be separated using various isolation methods, including ultracentrifugation, ultrafiltration, immunoaffinity capture, polyethylene glycol method, and others. For example, the filtered supernatant can be centrifuged with an ultracentrifuge (Beckman Coulter) at 100,000 g for 90 min. After that, the supernatant is discarded, and the exosome pellet is dissolved in ice-cold sterile-filtered PBS buffer and then stored at -80 °C until further analysis [[30], [31], [32],^36^].

## Exosome isolation methodologies

Exosomes' small nanoscale size and low buoyant density make them difficult to isolate and purify from complicated biological samples. Many different EV separation techniques have been discussed in the current review on the biological activity of exosomes. Therefore, the ability to efficiently and consistently isolate exosomes among several types of cell debris and other EVs is crucial. Depending on their size and affinity, multiple isolation methods could be applied to separate exosomes from biological fluids or cell culture supernatants. Typically, EVs are isolated according to their size, density, surface charge, or molecular composition of the membrane. The development of EV isolation techniques has been the focus of massive research in recent years [14]. Nowadays, the most commonly used separation methods are ultracentrifugation (UC), size-based chromatography (SC), immunoaffinity-based capture (IAC), polymer-based precipitation (PEG), and microfluidics-based (MF) approaches (Fig. 1).

### Ultracentrifugation methods (UC)

**Fig. 3:**
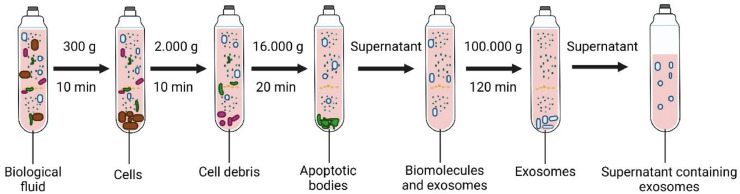
Ultracentrifugation method for isolating exosomes from biofluids.

The most commonly applied exosome separation method is UC, regarded as the gold standard, which is employed in 60% of exosome processing and is the most effective way to isolate exosomes from different biological samples [29]. The traditional UC method is based on the principle of sedimentation to isolate exosomes from complex biological samples such as blood serum or plasma, breast milk, cerebrospinal fluid, amniotic fluid, urine, aqueous humor, and cell culture lines [37,38]. It entails a series of low-speed centrifugations to separate cells, microvesicles, and apoptotic bodies, followed by high-speed ultracentrifugation at a speed of 100,000x g to precipitate exosomes [39], as shown in Fig. 3. All steps of centrifugation are maintained at 4 °C, and the pellet of exosomes can be resuspended in 300 to 500 µl of Dulbecco phosphate-buffered saline (DPBS) stored at -80 °C for subsequent analysis.

Ultracentrifugation is simple to perform, cost-effective over time, and allows large sample quantities [40,41]. However, high shear forces during ultracentrifugation can potentially disrupt the exosome structure. Exosome loss, fusion, deformation, and co-isolation of contaminants such as proteins are the key disadvantages of this method [42,43]. The UC method is categorized into differential ultracentrifugation and density gradient ultracentrifugation.

#### Differential ultracentrifugation (DUC)-timing:∼12 h

Differential ultracentrifugation is often called ultracentrifugation. Multiple rounds of centrifugation are used in the ultracentrifugation process known as differential centrifugation (differential velocity centrifugation) to isolate exosomes from other vesicles, proteins, and cell debris [44,45]. Exosomes are separated based on the sedimentation coefficient (S), which is proportional to their size and density. The process necessitates regular user involvement to separate pellets and supernatants and to establish spin cycles. Exosome loss might occur when the supernatant is repeatedly removed and the sample is transferred between tubes; as a result, exosome loss is expected, and higher sample quantities are required to be employed at the beginning of the process to achieve the target yields [46].

Differential centrifugation is most frequently used to isolate exosomes and is carried out at gradually rising speeds. This technique's key concept is the centrifugation-based removal of cell debris and large vesicles. First, biological fluid samples are centrifuged at 500, 3000, and 16,000x g (up to 1 h) to pellet cells, large debris, apoptotic bodies, and aggregates of biopolymers. Lastly, the exosomes are recovered in a pellet from the supernatant using a centrifuge at 100,000–150,000x g for 1–6 h. The pellet of exosomes can then be washed and resuspended in sterile-filtered phosphate-buffered saline (PBS) buffer for subsequent analyses or long-term storage at -80 °C for further analysis. In a study by Kowal et al., it was discovered that vesicles with a diameter between 50 and 150 nm make up 70% of the exosome fraction obtained using this technique, with the remainder of vesicles having dimensions higher than 150 nm or less than 50 nm (20%) [47]. This method is labor-intensive, depending on the rotor type and its parameters, the beginning liquid's temperature, and viscosity. It requires customizing conventional centrifugation procedures based on the rotor employed and the characteristics of biofluid samples [48]. The main advantages of this approach are its inexpensive processing cost, its ability to deal with excessive amounts of samples (1.0 ml to 25 ml) and separate several EV samples at once, and the lack of extra chemicals required for this procedure. Due to different vesicles with similar sizes and protein aggregates that can co-form at 100,000 x g, this method may not be suitable as the biological fluid has a high degree of heterogeneity. Also, centrifugation at high speed with a long spinning period may induce aggregation of exosomes.

#### Density gradient ultracentrifugation (DGUC)-timing:∼24 h

To facilitate exosome isolation between various media layers, density-gradient ultracentrifugation (DGUC), also known as isopycnic ultracentrifugation, employs several previously established discontinuous density layers [14]. This is contrary to differential centrifugation which uses multiple extended high-spin cycles using zonal ultracentrifugation [49]. It is well known that DGUC centrifugation makes it possible to separate subcellular components and improves particle separation effectiveness based on their buoyant density [40]. Exosomes are separated by DGUC centrifugation according to the size and mass density differences between them and other components.

A wide range of samples, such as blood, cell culture, breast milk, saliva, and urine, have been extensively employed with DGUC centrifugation. For instance, exosomes have been extracted via DGUC centrifugation obtained from saliva, which contains a combination of cell debris, gingival crevicular fluids, gland secretions, microorganisms, and cell debris [50]. The particles move across the gradient during the centrifugation process until they reach a point where their density is the same as that of the surrounding fluid. Three typical media for making the gradient are sucrose, iodixanol, and iohexol. Exosome-containing samples can be loaded as "bottom-up" or "top-down" and then ultracentrifuged. Finally, the fractions must be carefully collected after separation by a gradient to avoid disrupting the gradient.

Choi et al. proposed a different technique for the separation of exosomes and downstream proteome analysis employing sample bottom loading [51]. In this procedure, samples are introduced to 0.8 and 2 M sucrose cushions before ultracentrifugation is carried out. After doing this twice, exosomes are discovered at the junction of two layers of sucrose cushion buffer. Compared to sucrose, iodixanol (OptiPrep) has several advantages including higher stability, lower viscosity, and better biological activity [52]. Iodixanol from OptiPrep (MilliporeSigma, US) is used in a density-based separation technique described by Ter-Ovanesyan et al. that makes use of sample top-loading [48,49,53]. After that, crude exosome pellets were dissolved in sterile-filtered PBS buffer and top-loaded into an iodixanol gradient solution.

To generate an OptiPrep (iodixanol) gradient for DGC, layers are prepared from bottom to top: 3 ml 40%, 3 ml 20%, 3 ml 10%, and 2 ml 5%. Briefly, the iodixanol gradient is prepared, using OptiPrep™ stock solution (60% w/v) diluted with 0.25 M sucrose/10 mM Tris, pH 7.5 to make 40%, 20%, 10%, and 5% w/v solutions. One milliliter of plasma is loaded to the 5% top fraction. Samples are then ultracentrifuged in polypropylene tubes at 100,000 rpm for 18 h at 4 °C. Fractions are then taken from the top in 1-mL increments. To determine the density of each fraction, 1 ml of sterile-filtered PBS is placed into a gradient rather than a sample. Following ultracentrifugation, the refractive index of each fraction is measured with a refractometer, and the density of the mixture is estimated. Following gradient separation, positive fractions are collected carefully to prevent interrupting the gradient, diluted to 10 mL in PBS, and ultracentrifuged again at 100,000x g for 2 h at 4 °C to reduce the background protein contamination. The pellets of exosomes are then resuspended in 100–200 µL of sterile-filtered PBS buffer and stored at -80 °C for subsequent analyses [19,54].

Building the polymer density layers adds to the cost and labor intensity. Due to these factors, large-scale density-gradient ultracentrifugation is mainly not recommended for exosome isolation. When comparing the density gradient ultracentrifugation to differential ultracentrifugation, isolation from the density gradient takes longer processes time with lower yield but produces exosomes with a better degree of purity [41,55,56].

### Size‑based techniques-timing:2–4 h

The three primary categories of size-based methods are ultrafiltration, sequential filtering, and size-exclusion chromatography.

#### Ultrafiltration (UF)

Ultrafiltration (UF) also named membrane filtration, which has a molecular weight cutoff (MWCO) value of 10, 50, and 100 kDa, is employed as a basic step for extracting exosomes from vast amounts of original material, such as cell culture, into concentrated small volumes that can be used in additional purification processes or analysis [57]. The concept behind this method is that exosomes can be isolated from all other sample components by using membranous filters depending on their sizes and molecular weights.

**Fig. 4:**
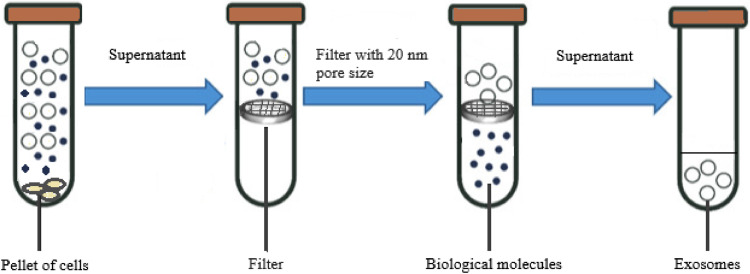
Ultrafiltration method to isolate exosomes.

In the first step of the procedure, exosomes are separated from larger contaminants including cells, debris, and microparticles using membrane filters with 0.1, 0.22, and 0.45 μm pore diameter. The soluble and aggregated proteins are then separated using commercial membrane filters for example, the Corning Disposable Bottle-Top Filter or the Amicon Centrifugal Filter, which have molecular weight cutoffs ranging from 5 to 100 kDa. If further volume reduction is required, samples might be centrifuged between 100,000 to 200,000 x g for pelleting exosomes (Fig. 4).

Ultrafiltration separates exosomes using pressure to push sample fluid through membranes having pores smaller than 100 nm [14]. More processes can be employed to filter out additional undesirable particles using membranes with nanoscale or larger pore diameters. The procedure is more rapid than ultracentrifugation; however, the pressure used may cause exosome damage due to shear stress, exosome loss because of membrane adhesion, and membrane blockage from particle aggregation, which may decrease exosome yield and increase processing time [14,58]. Sequential filtration, centrifugal ultrafiltration, tandem filtration, and tangential flow filtration are a few types of exosome ultrafiltration methods [14,59].

Tandem filtration combines numerous filters in a single syringe, while sequential filtration involves multiple rounds of filtering, each with a different molecular weight cutoff. The sample content is forced through a nanoporous membrane that is attached inside a tube by centrifugal force as the membrane is rotated within a tube [60,61]. The removal of large particles from samples including cells, intact organelles, apoptotic bodies, and protein aggregates, as well as clogging prevention, is commonly accomplished by preparatory centrifugation or dead-end filtering at 0.22 μm before centrifugal ultrafiltration.

Recently, tangential flow filtration (TFF) has also been utilized to isolate exosomes with higher yields [59]. Unlike the abovementioned methods, TFF passes samples tangentially to the membrane rather than applying pressure orthogonally [61]. The limitations of this technology include exosome damage and contamination by solution components that are smaller in size than the pores of the filters. Moreover, some exosomes may be absorbed into the membrane, meaning that some exosomes would be lost, which is important for extracting tiny amounts of biological fluids. Compared to ultracentrifugation methods, TFF is gentler to the sample and can process larger quantities of fluid with improved reproducibility. However, compared to other filtration techniques, TFF takes more time to process. Exosome ultrafiltration is less complicated and faster and requires less specialized equipment than ultracentrifugation isolation.

#### Sequential filtration

Typically, there are three steps in the sequential filtration process. Cell debris is filtered in the first phase, free proteins are eliminated, and exosomes are separated using filters with suitable pore diameters [62].

#### Size exclusion chromatography (SEC)

**Fig. 5:**
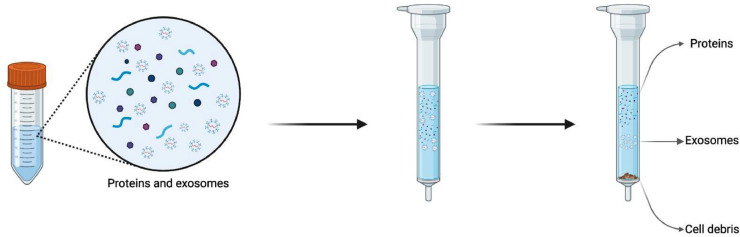
Size exclusion chromatography (SEC) separates exosomes from biological fluid samples.

SEC, also named gel filtration, the gentlest chromatography method, is frequently applied to isolate and purify particles including exosomes, according to their size. Exosome isolation using SEC gives a high yield while maintaining vesicle integrity and biological function [63]. By using this SEC method, samples are placed on the top and passed throughout a porous stationary phase of a chromatography column; those components of a mixture with a small hydrodynamic radius can elute more quickly through the porous pore material including agarose (Sepharose), polyacrylamide (Sephacryl), and dextran polymer (Sephadex) as they can pass through them more rapidly. In contrast, exosomes with larger radii are blocked from passing the pores [64,65]. Processing characteristics, for example, column dimensions, type of resin, bead packing, flow rate of the mobile phase, and system volume are crucial to consider when obtaining high-resolution particle sizes [66]. Although it is completed quite quickly, ultracentrifugation is needed in a subsequent step to concentrate the material [41]. It effectively eliminates impurities such as lipoproteins and plasma proteins, but it is challenging to obtain a pure exosome [41] (Fig. 5).

Pretreatment of samples using ultracentrifugation or ultrafiltration is required to produce EV preparations devoid of protein and other biomolecule impurities [67]. There are several commercially available prepacked columns, including qEV (Izon Science) and HiLoad Superdex (GE Healthcare), to make EV isolation by SEC easier [68]. SEC with prepacked columns yields a lower exosome extraction efficiency rate and a more heterogeneous EV population than precipitation-based isolation. Still, it is quick, accessible, repeatable, applicable to distinct types of samples, and does not need a chromatography system since it may be used with a separate pump system. The advantages of SEC over centrifugal and filtering techniques are numerous, including cost-effectiveness, reproducibility, and non-destructive results.

Notably, this technique is also appropriate for exosome isolation from blood plasma and serum [23]. Meanwhile, this technique can be applied to separate materials with different viscosities, ranging from low-viscosity cell culture medium to high-viscosity plasma. The biophysical and functional characteristics of the isolated vesicles can be preserved while high-yield isolation is produced via size-exclusion chromatography [[69], [70], [71], [72]]. Although vesicle deformation and rupture issues have been noted, it is possible to minimize them by choosing the right stationary fractionation column and carrying out chromatographic separation using only gravity. This method is acknowledged as being effective for isolating EVs. In contrast to UC, SEC can maintain isolated exosomes' biological activity and integrity. In addition, SEC can be used with a sample of as little as 20 μl and the entire procedure can be completed rapidly (10 to 20 min) [26,73]. However, exosomes, microvesicles, protein aggregates, and lipoproteins of the same size cannot be effectively differentiated by the SEC method which results in low purity, and the specialized equipment and columns used are also expensive.

### Immunoaffinity-based capture (IAC)-timing: 1–2 days

Exosomes are isolated in immunoaffinity-based capture methods (IACs) using certain antibodies or affinity ligands coated on magnetic bead surfaces to target specific receptor proteins of the exosomes. Magnetic beads like iron, magnetite, neodymium, or nickel coated with antibodies have the potential to be easily functionalized with exosomal receptor antigens. This methodology enables the study of variations in the functional consequences of exosome subtypes by allowing the separation of diverse exosome subpopulations produced by different cell types. Additionally, this method enables the imaging of individual exosomes and the identification of protein markers on their membranes. IAC is a gentle procedure that maintains the function of exosomes following their separation and purification [74].

It is crucial to understand that immunomagnetic beads, a unique tool that can be altered to attach to target receptor proteins on the membrane surfaces of exosomes, play a significant role in capture-based methods. Exosomes' surfaces contain a variety of membrane proteins, including Clusters of Differentiation (e.g. CD9, CD56, CD63, CD81, CD82, CD91, CD105, CD147, and CD151), Apoptosis-Linked gene 2-interacting protein X (ALIX), and Epithelial Cell Adhesion Molecule (Ep-CAM), which can be enhanced using magnetic beads with the corresponding antibody coatings [49,61,75].

**Fig. 6:**
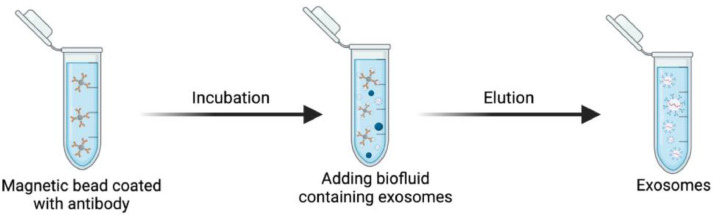
Immunoaffinity-based method. Magnetic beads coated with antibodies against receptor proteins on the membrane surface of exosomes.

In this approach, the samples are incubated with certain antibody-coated magnetic beads, and then exosomes isolated from the rest of the biomolecules in the sample are captured using a magnetic field [65,76]. Then, the magnetic beads are washed to reduce the nonspecific interactions and unbound materials, and immobilized exosomes are then collected in a stationary phase, depending on the specific immunological interaction between the antibodies and target receptor proteins of the exosome. By changing the buffer's composition, adding excessive target molecules (like sugars or lipids), or removing molecules necessary for effective binding (such as chelating calcium with EDTA), bounded exosomes can be separated from the matrix and recovered. This method satisfies the strict requirements for isolating exosomes that contain certain target receptors of their membrane. To eliminate protein aggregates and other large particles, immunoaffinity capture is frequently combined with additional preprocessing techniques such as SEC [77] or centrifugation [78] (Fig. 6).

The specificity of exosomes extracted with immunoaffinity is significantly higher than that of ultracentrifugation [40,79]. However, some studies indicate that exosome yields from immunoaffinity separation are lower than those from other techniques because some biomarkers may not be present or recognized on some exosomes [80]. Immunoaffinity techniques have two main drawbacks: they can only be used with lower sample volumes and take a long time to complete when used as a further purification step of another isolation technique. Additionally, this approach can only isolate subtypes of exosomes with positive markers, not all types of exosomes [79]. Altogether, the need for specialized equipment, the expensive cost of high-affinity antibodies or ligands to immunocapture particular exosomes, nonspecific binding, and time-consuming are some of the limitations of this method.

This approach has a lower yield because of sample volume limitations that may be analyzed and are limited by the availability of antibodies [74]. Furthermore, a lengthy incubation period is necessary. For instance, Thermo Fisher's magnetic Dynabeads, which are frequently employed, need two 12-hour incubation periods: one for antibody conjugation and the other for bead capture [77]. These prolonged incubation times are required because of the large bead size (1.0–4.5 μm), limited mobility in solution, and lower surface area to total volume amount [60].

Magnetic or antibody-coated latex beads are typically used to incubate the sample [41]. It is possible to catch exosomes carrying such surface antigens by coating magnetic beads with certain antibodies. For instance, magnetic beads coated with CD56 or CD171 antibodies have been used to catch exosomes originating from neuronal cells [81,82]. In contrast, chondroitin sulfate peptidoglycan 4 antibody-coated beads have been used to capture exosomes derived from melanoma cells [77]. Exosomes generated from a particular cell type may be isolated using additional biomarkers. Using magnetic nanoparticles that are temperature- or pH-responsive is one way to speed up this process [60]. Due to the nanoparticles' significantly higher surface area to volume ratio (40x greater for 25 nm nanoparticles than for 1 µm Dynabeads) and greater magnetophoretic mobility, which enables rapid magnetic separation after aggregation caused by temperature changes or pH, this method reduces the incubation and isolation periods to only just a few minutes. Immunoaffinity methods based on Raman scattering also utilize magnetic characteristics to separate and characterize exosomes. Molecules can be recognized by Raman scattering using their unique chemical fingerprints. The immunoaffinity technique is useful for isolating a particular subpopulation of exosomes that originated from a specific type of tissue cell due to its high specificity and purity. Breast cancer has been successfully identified in patient samples using magnetic surface-enhanced Raman scattering, which has great sensitivity and specificity [83]. This method differs from conventional immunoaffinity approaches in integrating characterization with sample processing, which is essential to simplify the exosome applications in therapies and diagnosis.

### Precipitation techniques -timing:∼2 h

**Fig. 7:**
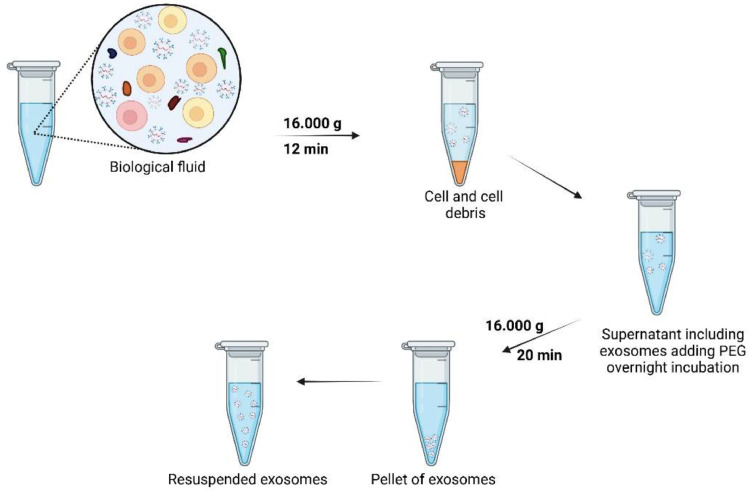
Precipitation method to isolate exosomes from biological fluid samples.

Contrary to the isolation mentioned in earlier procedures, precipitation methods primarily rely on employing highly hydrophilic polymers to precipitate exosomes chemically. The most widely used polymer for exosome separation is the highly hydrophilic polymer polyethylene glycol (PEG) which strongly encourages enrichment and boosts exosome yield [84]. Exosome precipitation relies on the use of polymers such as PEG (MW: 8 kDA) that bind to water molecules surrounding the exosomal membrane due to their negative charge [85]. PEG pulls water molecules in and pushes less soluble particles out of the exosome. This method involves overnight coincubation of samples with 8–12% of 6‑kDa PEG solution at 4 °C. It has been established experimentally that the addition of positively charged protamine molecules may stimulate exosome aggregation during the incubation period [86]. Adding a neutralizing substance, such as cationic beads, the complex can be precipitated and processed further using several separation procedures, including filtration and low-speed centrifugation (Fig. 7). The PEG method has recently been applied in clinical applications and is incorporated into various commercially available nanoparticle formulations, including the novel mRNA-based COVID-19 vaccines and the chemotherapeutic liposomal drug Doxil [87].

In 2017, Helwa et al. evaluated ultracentrifugation and three commercial precipitation kits using PEG for isolating exosomes from serum: ExoQuick-Plus (System Biosciences, USA), Total Exosome Isolation Reagent (TEIR; Invitrogen, USA), and miRCURY Exosome Kit (Qiagen, USA) [37]. Compared to commercial kits, the number of exosomes recovered by differential centrifugation was significantly less (up to 130-fold, depending on the initial volume). The yields of all commercial kits were comparable, apart from TEIR, which had a significantly higher yield than miRCURY. The samples used in experiments are precleared to eliminate cells and cellular waste, according to the ExoQuick user manual. Depending on the kind of sample, the cleared solution is subsequently incubated for 0.5–12 h with the proper volume of ExoQuick. The last step is to centrifuge exosomes for 20 min at 16,000 x g at 4 °C. The pellets of exosomes are then resuspended gradually in the proper sample buffer such as sterile-filtered PBS and kept at -80 °C for subsequent analysis. Several companies have created rapid and simple exosome isolation kits to reduce the time and sample volume restrictions of the standard procedures; nevertheless, the reliability and specificity of these kits can be varied, and they are not always the most cost-effective option.

The precipitation-based isolation method is easy, rapid, inexpensive, necessitates a small sample size, and does not require specialized equipment. Biomaterials are concentrated in these polymers' solvent regions until their solubility is surpassed, at this point, precipitation occurs. Precipitation is known to be the simplest and fastest method to isolate exosomes, as it does not destroy them and does not require any additional equipment. These approaches are the most appealing for clinical research. Although this process produces a larger yield, a disadvantage is the lower product purity [88,89]. It has also been noted that these techniques suffer from the simultaneous removal of several contaminants from the sample, such as non-exosomal proteins and other particulates [90].

As a result, the use of precipitation methods to analyze exosomes from biological fluid samples is restricted by high protein contamination. Additionally, exosomes separated via precipitation methods may contain biopolymers that can complicate subsequent analyses, such as proteomic analysis, RNA assays, and mass spectrometry. To reduce contamination with non-exosomal contaminants from the samples, an effective pre-filtration step can be added using a 0.22 μm filter or a post-precipitation purification process that includes subsequent centrifugation, filtering, or gel filtration [91]. Due to ongoing research, the current polymer precipitation process has been upgraded and improved throughout time, significantly improving the exosome isolation from human biological fluids. ExoQuick Plus (System Biosciences, USA) and ExoEasy (Qiagen, The Netherlands) are two commercial kits that have been developed currently to employ polymer precipitation and can produce exosomes with a high yield and purity compared with previous kits [92]. In conclusion, whereas commercially available kits offer a convenient substitute for rapid and labor-saving exosome separation, their current lack of specificity must be resolved before they can be employed regularly.

### Microfluidics‑based techniques (MF) -timing:∼24 h

One of the newest methods to isolate exosomes from small volumes of biological fluids is the signal detection-based microfluidic method. Exosome isolation from other nanometer-sized particles can be accomplished with the use of microfluidics systems since they facilitate rapid, accurate, and cost-efficient isolation procedures [93]. Currently, size-based, immunoaffinity-based, and dynamic separation are all fully integrated into widely used microfluidics tools. The exosome total isolation chip (ExoTIC) device was unveiled in recent decades as a new exosome isolation technology. The ExoTIC system provides a higher yield, purity, and efficiency during exosome isolation from serum or other physiological fluids than PEG precipitation and ultracentrifugation [94].

Microfluidic systems are referred to as integrated systems with two or more devices that are assembled to operate in parallel autonomously. Typically, one or more devices comprise a network of interconnected microchannels that can manage lesser amounts of media [95,96]. This capability enables microfluidic devices to reproduce complex analytical procedures on a microscale, with high specificity and precision. Then, further specialized components can be included to facilitate fluid movement or increase the number of available analyses [97].

Most immunoaffinity-based microfluidic exosome isolation techniques use similar procedures, which entrap analytes using a general lateral flow assay design [98]. In this procedure, the base of the microfluidic device such as the ExoChip (fabricated in polydimethylsiloxane) is coated with antibodies against commonly overexpressed exosomal surface markers like CD9, CD63, and CD81 which binds the exosomes and allows for their separation, collection, and analysis [99]. When a sample of exosome-containing biomarkers passes through a chip, the exosomes are captured and retained by binding agents while other molecules flow through. Microfluidics devices have used arrays of silicon nanowire micropillars that use size exclusion to capture exosomes [100]. In this system, exosomes are trapped in openings as fluid flows through the device. Although this procedure's first step is fast, releasing exosomes from the pores can take 24 h. Because of this time-consuming step, the efficacy of this process for diagnostics is limited.

**Fig. 8:**
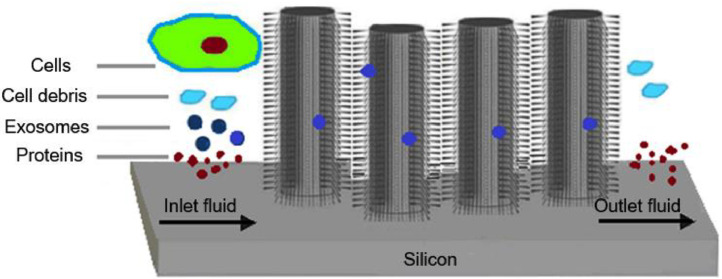
Microfluidics-based technology for exosome separation from biological fluid samples.

The viscoelastic-based microfluidic platform has also been used to achieve exosome separation by size [[101], [102], [103]]. In this platform, samples are combined with biocompatible polymers that are elastically responsive before being put into viscoelastic media. Larger particles including cells, cell debris, and microvesicles with higher elastic pressures from serum or cell culture media are pushed away from the exosomes as the fluid moves through the device (Fig. 8). Recently, Meng et al. developed a viscoelastic microfluidic platform to enable the continuous and label-free separation of exosomes directly from blood samples of cancer patients. A cell-depletion module to eliminate blood cells, and medium and large vesicles (lEVs and mEVs). They discovered that the apparatus successfully isolated exosomes with a diameter of ∼100 nm, exhibiting 97% purity and 87% recovery rate at a 200 μl/h sample volumetric flow rate [103].

On the other hand, exosomes can be separated according to their size using an acoustic microfluidic method [99,104]. Acoustic waves are gentler and require less touch compared to micropillar arrays. Interdigital transducers use a flowing sample to produce waves everywhere over it. The wave frequency determines the particle size cutoff at the two channels for separation. The waste from one channel includes apoptotic bodies and large microvesicles, whereas the waste from the other channel only contains exosomes with a purity of ∼98%. This model requires about 25 min to complete the running of each sample, and because there is no interaction, exosomes retain their biological function.

Microfluidics has also employed electrical waves in addition to acoustics to separate exosomes in a label- and contact-free manner [105]. The more negatively charged exosomes compared to other particles are taken advantage of in ion-based separation [106]. Positively and negatively charged particles are separated using a microfluidic device modified by Mogi et al. that has two inlet channels and two exit channels with high and low-voltage [106]. In this apparatus, positively charged particles are drawn to the channel with low voltage whereas negatively charged particles are drawn to the channel with high voltage in each pair. Uncharged particles stay close to the center and are pushed into the channels by an ion depletion zone created by a perpendicular ion channel in the center. This apparatus has shown a noticeably higher yield than ultracentrifugation and is calibrated for voltage and flow rate to enhance the retention of exosomes.

The flowchart's characterization section includes the topology, single vesicle, qualitative, and quantitative characterization. The procedure begins with determining and recording the initial material properties, including cell count, fluid volume, and nonexosomal molecule quantity. Next, a processing technique that will produce the desired yield and purity is chosen based on the starting material. However, the application of microfluidics-based technologies for isolating exosomes has some disadvantages, including the need for specialized expensive equipment, low sampling efficiency, frequent channel blockage, and the requirement for high affinity and specificity antibodies.

#### Comparisons of exosome isolation techniques

Purity and yield measurements of nucleic acids, proteins, lipids, and other particles are the first step in exosome characterization after isolation. Additional processing is needed if the sample does not meet the requirements for yield and purity. Table 1 summarizes the results of comparative studies of common exosome isolation methods [68,91,[107], [108], [109]] in terms of exosome yields and purities. Yields were ranked according to particle counts per unit volume, and purity was measured by dividing the total protein content of a solution by the number of vesicles present [68,107].

**Table 1:** Comparison of various exosome isolation methods.

Methods	Advantages	Disadvantages	References
Ultracentrifugation techniques (UC)	Most used and well-developed, Large volumes can be used, Multiple samples can be isolated at the same period,	Expensive equipment (ultracentrifuge), Presence of contaminants, Time consuming, labor-intensive, Exosome damage due to high-speed centrifugation, Low purity and yield recovery,	[[37], [38], [39],^68^,^114^]
Differential centrifugation	Large volumes can be used,	Presence of contaminants, Time-consuming,	[44,46,47,108]
Density gradient centrifugation	High practicability, Standardized protocol, Potential for EV subtype isolation,	Labor intensive, Excessive cost and time-consuming, It may be required to remove the gradient material,	[41,52,56,115,116]
Size‑based techniques	Easy to perform,	Presence of contaminants,	[61,62]
Ultrafiltration	Fast and easy to perform, Size uniformity of yield, The most often utilized as the first stage of cleanup or as the post-isolation concentration step,	Low yield, High pressures may damage the membranes of larger EVs, Membrane pores can be blocked when filtering large volumes, Low purity,	[14,[57], [58], [59], [60]]
Size-exclusion chromatography (SEC)	Economical and nondestructive, Low price, Efficient at removing small proteins, High scalability, Maintains structural integrity and biological activity,	Complicated, Labor intensive, Time consuming, Contaminants of a matching size of EVs may coisolate,	[41,63,64,67,[69], [70], [71], [72]]
Immunoaffinity capture-based isolation	High purity and specificity, Easily available commercially, Special equipment is not required, Small sample volumes can be applied,	Low yield, Nonscalable, Separate exosomes with targeted proteins only, The surface proteins and functioning may be damaged during elution, There is no universal marker for exosomes, Costly antibodies are required and difficult to remove afterward,	[41,63,64,67,[69], [70], [71], [72]]
Precipitation techniques	Commercial kits, Easy to use and very rapid, Convenient operation, High yield, Large volumes can be used, Special equipment is not required,	Unstable quality of kits, Presence of contaminants, Low purity,	[84,85,[88], [89], [90]]
Microfluidics‑based techniques	Fast separation, continuous process, High purity, Very small sample volumes can be applied, Allow exosome isolation and characterization simultaneously,	Low sample capacity, Complicated equipment, difficult to operate, Required trained personnel, Excessive costs in device development,	[[93], [94], [95], [96],^117^]

Exosome separation from platelet-free plasma was utilized as a model system in the comparative study by Tian et al. to assess exosome yield and purity when using various commercial products [107]. In this study, precipitation-based kit ExoQuick (System Bioscience), Total Exosome Isolation kit (TEI; Thermo Fisher), SEC-based qEVsingle kit (IZON Science) as well as ultrafiltration equipment (Millipore) were all put to the test. For reference, ultracentrifugation was also used during this study. As a result, the highest yields to the lowest yielding techniques were ExoQuick, TEI, ultrafiltration, SEC-based qEVsingle, and ultracentrifugation. By comparing precipitation with other methods such as ultracentrifugation, sucrose density gradient with ultracentrifugation, and size-exclusion chromatography, Ludwig et al. found that the precipitation method gives a higher yield but with lower purity compared to other techniques [68].

Patel et al. compared the efficiency of the exosome isolation method using four commercial kits that use various isolation techniques: total exosome isolation kit (TEI, Thermo Fisher), precipitation and filtration combination (the PureExo Exosome Isolation kit from Fisher Scientific), immunoaffinity capture (MagCapture, Fujifilm Wako), and SEC (qEVsingle, iZON Science) [108]. In this study, immunoaffinity capture demonstrated greater purity but lower yield than the other three approaches. In contrast, the precipitation combined with filtration (PureExo) produced a higher yield and purity of exosomes isolated from the culture supernatant.

Purification of urinary exosomes was carried out by Alvarez et al. using a variety of techniques, such as traditional ultracentrifugation, sucrose density gradient ultracentrifugation, filtration with ultracentrifugation (0.22 μm filter, Millipore), ultrafiltration (Vivaspin 20, Sartorius), and precipitation (ExoQuick, System Bioscience) [109]. The research demonstrated that ExoQuick precipitation with a larger amount of ExoQuick-TC reagent and faster final centrifugation speed is a suitable alternative to a larger number of samples if urine specimens are preprocessed with dl-dithiothreitol, and purity might be improved by adding SEC process. Using human plasma and cell culture medium as initial materials, Lobb et al. examined several isolation methods. According to the results of their study, repetitive ultracentrifugation may damage vesicles [91]. In contrast, combining ultrafiltration and SEC increases the yield and purity while reducing the processing period. In a recent study, Kapoor and colleagues created a novel technique using Size Exclusion Fast Performance Liquid Chromatography (SE-FPLC) that allowed for the efficient as well as rapid isolation of EVs from a variety of biofluids and cell line models, resulting in higher yield and purity. Their research demonstrated that the SE-FPLC has the potential to advance EV research in the life sciences and speed up its translation into clinical trials [110].

Ansari and colleagues conducted a comparative study to evaluate the effectiveness of ultracentrifugation, ultrafiltration, and precipitation techniques in isolating exosomes from cardiomyoblast H9c2 cell culture. They reported that the exosomes isolated by the ultracentrifugation method have higher purity, homogeneity, and functionality than the sample isolated with the ultrafiltration and polyethylene glycol precipitation methods [111]. In another study, Williams et al. compared ultracentrifugation (UC), polyethylene glycol (PEG) precipitation, ultrafiltration (UF), size-exclusion chromatography (SEC), aqueous two-phase system (ATPS), and total exosome isolation reagent (TEIR) methods for isolating exosomes from a murine skeletal muscle myoblast C2C12 cells. Their findings indicated that SEC and UC recovered exosome-enriched fractions with the highest levels of exosome biomarkers, including CD9, CD63, CD81, ALIX, and annexin A2 in Western blotting analysis [15]. In addition, in two recent studies, exosomes were separated from human plasma using five different isolation techniques: SEC, DUC, DGUC, PEG, and IAC. The results demonstrated that SEC-processed samples yielded higher quantities of proteins and exosomes with sufficient purity than the other four methods [112,113].

Recently, Li et al. developed a rapid (<30 min) method for isolating exosomes using reversible zwitterionic coordination between exosome membranes and "PC-inverse" choline phosphate coated on magnetic beads [102]. This method isolates exosomes from many biofluids, including blood, saliva, and urine with a yield and purity of over 90%, outperforming traditional exosome isolation methods regarding speed, simplicity, yield, and purity.

#### Summary of exosome isolation methods

The exosome origin, amount, and aims of the subsequent analysis all play a significant role in choosing the best isolation method. The most crucial factors to consider when selecting the optimal exosome separation method for a type of research are (1) starting sample type and equipment availability; (2) the exosome yield and collection rate of the process; (3) the obtained exosome sample's purity; and (4) the efficiency of the methodology in terms of effort and time. In summary, an ideal method for isolating exosomes should be easy to use, rapid, effective, reasonable, low cost, high purity, high yield, and accessible. Furthermore, it should not affect the natural form of exosomes or require expensive equipment. Different methodologies have specific advantages and disadvantages regarding efficacy, repeatability, and influence on functional results. These limitations might be solved and exosome studies for both fundamental and therapeutic applications advanced through further protocol modification and combining two or more isolation methods, but there are still restrictions.

#### Recovery rate

According to numerous studies, PEG-based precipitation has a recovery rate of approximately 80–90%, which is higher than the other methods [[118], [119], [120]]. Due to the loss of a sizable portion of exosomes during the procedure, numerous studies have demonstrated that differential centrifugation only has a recovery rate of 20–40% [20,91,119,120]. Compared to differential centrifugation, ultrafiltration has a higher recovery yield, at approximately 60% [81,99]. Both ultrafiltration and differential centrifugation are frequently employed in studies, and the selection to utilize one over the other is typically based on personal preferences in the laboratory. Since PEG-based precipitation offers the highest percentage of exosome yield recovery, it is frequently utilized in trials with small starting sample volumes, for example, those using clinical biofluid samples including blood plasma and serum, amniotic fluid, vitreous, CSF, and urine [121]. One of the recent studies for exosomal stability stated that the isolated exosomes could be kept for a long time without any significant loss of function or yield if kept at −80 °C in sterile-filtered PBS buffer including 25 mM trehalose [122].

#### Purity

Removing nonspecifically bound proteins from vesicles by density gradient ultracentrifugation is currently regarded as the ‘gold standard’ for producing the purest exosomes [118]. As a result, exosome isolation for exosomal proteomics and RNA profiling research is frequently carried out using density gradient ultracentrifugation [25,123]. Exosome aggregates are a significant artifact in differential centrifugation and ultrafiltration, even though both methods can produce samples with comparably important levels of purity. Exosome samples produced by PEG-based precipitation often have the lowest purity. Exosome products usually contain an unknown amount of exosome content and contaminants, including proteins, nucleic acids, viruses (exosome-sized), and vesicles from other sources, requiring further work. Consequently, improved methods and equipment are needed to increase separated exosomes' purity, quality, identity, safety, and stability.

#### Time and labor cost

Density gradient centrifugation needs the most time and work of all methods; the full procedure takes 2–3 days to complete. Ultrafiltration, membrane filters, and immunoaffinity supplies are more costly than the materials required for differential centrifugation if the basic laboratory equipment has been placed. PEG-based precipitation in exosome isolation requires lower cost, shorter time, and less labor than other techniques.

## Methods for exosome characterization

Exosomes isolated and identified by any of the abovementioned methods should then be quantified and characterized in size, morphology, surface charge, density, biomarker expression, yield, and purity. Flow cytometry (FC), resistive pulse sensing (RPS), dynamic light scattering (DLS), electron microscopy (SEM/TEM/Cryo-EM), atomic force microscopy (AFM), and nanoparticle tracking analysis (NTA) are examples of frequently used as characterization techniques (Fig. 1). Three essential considerations for all electron microscopies should be stated: fixing, adsorption, and negative staining procedures. Exosome characterization methods are based on exosomal morphology and biomarker protein composition. The common exosomal biomarker proteins are multi-transmembrane proteins (e.g., CD9, CD40, CD63, CD81, CD82, heterotrimeric G proteins GNA), single-transmembrane proteins (e.g., Integrins, Major Histocompatibility Class I and II, heparan sulphate proteoglycans, and transferrin receptor), and lipid-anchored proteins (e.g. CD73 and CD59) [76].

### Ultrastructural analysis

#### Transmission electron microscopy (TEM)

TEM is the most efficient instrument for examining exosome morphology and structure due to its ability to detect exosomes of a tiny size. The negative staining process is simple and quick, taking only a few hours, and the resolution for TEM is approximately 1 nm. Exosomes can be examined using regular TEM to (1) confirm their presence in the solution; (2) rate their quality; and (3) examine their morphology. The widely used TEM technique can also examine the size, morphology, and structure at the single exosome level and detect their impurities.

In TEM analysis, exosomes were briefly treated in 2% paraformaldehyde, placed on Formvar carbon-coated TEM grids, and incubated for 20 min. The exosomes are then incubated with glutaraldehyde, washed, air-dried, and stained with uranyl acetate [124]. TEM works on the same fundamental concepts as a light microscope, except it uses electrons instead of light. By exposing a tiny layer of a specimen with an electron beam, electromagnetic lenses can detect how the electrons scatter, creating a diffraction pattern that can be captured as a magnified image. Vesicular diameter, including exosomes, can be measured using TEM images. Under TEM, EVs have been discovered in various forms of morphologies, from spherical to cup-shaped [44,125]. Exosomes, unlike cells with a cytoskeleton, lack an internal support structure. For this reason, when an exosome sample is dehydrated for TEM investigation, the vesicle might form a cup shape and lose its original morphology [99,126].

The precise localization of particular protein molecules found in the lumen of EVs is one of the most critical aspects of understanding their biological processes. Biological samples could be harmed by the electron beam used. Protein molecules found in EVs can be labeled and visualized using certain fluorescent dyes. However, the excessive fluorescent signal from labeled proteins restricts the use of TEM in imaging labeled EVs [127]. A substitute method, immunogold TEM, is needed to visualize EVs bound to labeled antibody probes.

#### Cryogenic electron microscopy (Cryo-EM)

One type of TEM is cryo-EM which permits samples to stay in their natural aqueous environments compared to air-dried samples, as is done for SEM and TEM. In the cryo-TEM technique, no sample dehydration or fixation is necessary, and the sample is stored in liquid nitrogen (below − 175  °C) with no evidence of elemental redistribution or ultrastructural changes. Cryo-TEM is considered one of the best techniques for imaging biomolecules in biological fluids without dehydration artifacts. Cryo-TEM has also been applied for EV analysis to prevent damage from the electron beam. It is also appropriate for photographing EVs with membrane structures and lumens.

Exosomes in suspension are briefly placed on a grid for cryo-EM analysis, and the grid is quickly submerged in liquid ethane to enable the vitrification of the sample. Samples can be transported in liquid nitrogen for storage or examined under cryo-EM analysis after vitrification [128]. Exosomes occasionally include smaller vesicles surrounding them, as Yuana et al. demonstrated in their cryo-EM study [129].

#### Scanning electron microscopy (SEM)

SEM examines samples line by line using a fine point beam rather than a broad beam as TEM does. As a result, SEM concentrates on the surface of materials to provide a three-dimensional image of exosomes as opposed to the two-dimensional image produced by TEM. Exosomes are briefly fixed with glutaraldehyde and dehydrated with ethanol at ascending concentrations. Then exosomes are prepared for SEM examination when samples are air-dried at room temperature [50]. After that, the fixed samples are sputter-coated to create an imaging-enhancing thin layer of conductor material, such as carbon or gold. In contrast to the cup-shaped morphology detected under TEM, SEM revealed spherical, bulging exosomes without central depression [77].

#### Atomic force microscopy (AFM)

Exosome topography studies can be conducted using AFM, which has a resolution of approximately 1 nm. Using AFM immunogold imaging, Sharma et al. discovered many CD63 receptor protein binding sites on the exosomal membrane [77]. In this method, the exosome samples are briefly spread out on a mica surface and left to air dry at room temperature. After cleaning with ultrapure water, the samples are dried again using nitrogen gas. Antibody coating of the mica is an additional option for capturing exosomes with specific antigens on their surface for imaging. AFM with silicon or silicon nitride probes is used to observe exosomes, and AFM software is used for their analysis [130]. Several research studies have shown how well AFM works for analyzing different physio-chemical properties of EVs derived from biofluids such as blood and saliva. It recognizes the interaction between a probing tip and target exosome surface based on the principle of optical and electron diffractions.

This method has become a key tool for characterizing EVs' shape, abundance, biomechanics, and biomolecular makeup at the nanoscale [131]. It has expanded our understanding of EVs at the single-vesicle and subvesicular levels. In a noninvasive mode, AFM also measures samples in their natural states with gentle sample preparation. Different samples can have different native states, and analysis is then performed under various experimental settings, such as the condition of the used AFM tip, temperature, humidity, pressure between the probe and the sample, or altering scan speed, which could become the limitations of this method.

### Nanoparticle tracking analysis (NTA)

The advanced NTA system is one of the most applied techniques in EV research since it can be used to estimate exosome concentration and single particle size distribution. NTA has greater advantages compared to TEM and flow cytometry. In this system, 30–1000 nm vesicles are driven by a flow, and NTA software tracks the Brownian motion of individual particles and determines their morphological size and overall concentration [[132], [133], [134]]. The Stokes-Einstein equation (SE) and dynamic light scattering (DLS) are used in this method to investigate the particle size and exosome concentration. The observation and recording of fluorescent nanoparticles including exosomes in a solution are made possible by combining laser light scattering microscopy with a camera system.

In EV analysis, NTA tracks exosome motion by analyzing images of each particle. The direction of the EVs' motion is identified to calculate the particles' velocity, which is correlated with their sizes [135,136]. The NTA method detects EVs' morphological structure, concentration, and size distribution. Furthermore, NTA can examine various vesicles of all shapes and sizes with diameters as small as 30 nm. This property has been exploited to define the phenotype of vesicle subpopulations based on their recognition with certain antibodies or fluorescent markers [135,137,138]. When employing fluorescent NTA, it should be considered to report the total number of particles in the light scatter mode, the number of labeled particles in fluorescence mode, the label removal procedure, and a buffer/reagent control to examine labeling artifacts.

### Asymmetric flow field-flow fractionation (AF4)

The AF4 is one of the few technologies that can distinguish the multiple groups of extracellular vesicles, including tiny nanoparticles known as exomeres, smaller than exosomes. This method separates exosomes according to their density and hydrodynamic characteristics. Exosomes move along a forward laminar channel and are divided into various populations according to their Brownian motion. While larger particles appear to travel more slowly, smaller particles have faster overall movement and diffusion rates. For exosome quantification, AF4 and NTA have been compared in numerous studies. Unlike NTA, which in previous studies could only resolve a single broad peak between 50 and 150 nm, AF4 can distinguish between two distinct exosome subpopulations: large exosome vesicles ranging between 90 and 150 nm in diameter, and small exosome vesicles ranging between 30 and 90 nm in size [[139], [140], [141]]. As a result, AF4 is more adept at managing the size heterogeneity of exosomes and might be regarded as a cutting-edge analytical method for characterizing exosome subsets in biological fluids.

### Resistive pulse sensing (RPS)

In this method, vesicles are sized by RPS, using their electrical resistance as they move through a tiny aperture. Vesicles between 50 and 1000 nm in diameter can be detected using RPS. When examining particle size distribution, RPS has a higher size resolution and more accuracy than DLS and NTA. The exosome concentrations measured by RPS were closer to the TEM count than NTA. In contrast to RPS, which was unable to discriminate exosomes from cell debris, protein aggregates, or liposomes, Grabarek et al. found that exosomes assessed by NTA had a 5- to 10-fold higher concentration than RPS [142].

Recently, Young et al. developed RPS devices in the plane that included three nanopores in a series to estimate the exosome particle volume and diameter, two pore-to-pore areas for measuring electrophoretic mobility and zeta potential; and an in-line filter to prevent cellular debris and aggregates from entering the nanopores [143]. The nanopores in this model had 200 nm in wide and 200 nm in deep cross-sections, and exosomes ranging in width from 60 to 160 nm were easily resolved.

### Tunable resistive pulse sensing (TRPS)

TRPS, a novel non-optical technology, is currently being employed to quantify the size and concentration of extracellular vesicles. It has been successfully used to characterize colloidal particles and a wide range of nanoparticles and biomolecules in suspension, ranging in size from approximately 50 nm up to the cell size, a property crucial for examining cellular function and EV uptake [144]. However, this method has two significant drawbacks. The first is the system's stability, which might be compromised if the pores are clogged with debris of samples. The second requirement is sensitivity, which might be difficult to achieve if the target nanoparticles are too small to be distinguished from the measurement system's background noise. The tuning of system parameters including noise, sensitivity cutoff limits, and accuracy can address these problems up to a point [144]. This method has also received substantial research for EV size distribution assessments, and it has even been developed for delivering biomolecules to treat Alzheimer's disease and anti-oncogenic miRNAs to tumoral cells [145,146].

### Flow cytometry (FCM)

Exosome subgroups can be semi-quantitatively measured using the bead-based flow cytometry method described in several publications by looking for membrane biomarkers on their surface [[147], [148], [149]]. In brief, exosomes are bound to aldehyde/sulfate-latex beads using a 15-minute incubation period with continuous rotation. The process is terminated by adding glycine and bovine serum albumin (BSA) solution. Then, exosome-bound beads are washed in sterile-filtered PBS buffer and blocked in BSA solution. Primary and fluorescence-labeled secondary antibodies are sequentially added and compared with negative control to detect a certain membrane biomarker protein. Flow cytometry detects and examines the light scattered at the point when a liquid stream with suspended particles, including exosomes, meets a laser beam of a certain wavelength. Flow cytometry not only enables particle detection in the samples but also allows characterizing the structure and morphology of EVs [150].

Flow cytometry is highly suited for the repeatable analysis of clinical samples, in contrast to NTA, western blotting, and electron microscopy [151]. It is a potent instrument that enables the simultaneous multiparametric examination of up to thousands of particles each second (physicochemical characteristics including size, shape, and surface biomarkers). As a result, it is an effective method for measuring, categorizing, and purifying particles in suspension [42]. However, because background noise and particle light scattering overlap, standard flow cytometry cannot identify a sizable portion of particles (particularly exosomes). To solve these challenges, high-end, specialized flow cytometers have recently been developed with increased sensitivity, forward scatter detection (FSC), fluorescence amplification, and high-resolution imaging [152,153].

Imaging flow cytometry (IFCM), an alternate method to traditional flow cytometry, enables the identification of submicron-sized particles. The advantages of conventional FCM are maintained by IFCM, albeit at a higher resolution. IFCM has been demonstrated to be a workable approach for exosome analysis, enabling precise, straightforward, and high-throughput phenotypic assessment of exosomes [[154], [155], [156]]. Using photoacoustic (PA) signals in flow cytometry is another method for surface analysis. The analyte fluorophore is excited by a laser in traditional FCM and IFCM, but in PAFCM, the analyte is vibrated instead, producing a PA signal. Then, an ultrasonic transducer records these signals rather than using an image sensor. *In vivo* direct study of tumor-derived exosomes in mice was made possible by the combination of PAFCM and fluorescent FCM, allowing researchers to apply complementary detection peaks [157]. Although FCM is a valuable tool for tumor-derived exosome research, its failure to deliver precise, repeatable data severely restricts the study of exosomes. Recently, methods for EV detection by flow cytometry have been developed, focusing on standardization processes. Compared with other methods, flow cytometry has the significant benefit of detecting EVs as rare occurrences in large quantities, and by antigens on the surface, which identify their cellular origin.

### Fluorescence-based analysis (FBA)

In the various fields of life science study, fluorescence-based analytical systems have been thoroughly investigated. The configuration including magnification, laser power, and software of this optical system setup is always being improved to analyze EVs effectively. As a result of this approach, the background fluorescence from unbound dye is drastically reduced because the fluorophores are only excited within the light sheet. When compared to epifluorescence microscopy, the contrast is noticeably improved, and nanovesicles may be detected at a better resolution [158]. Fluorescence correlation spectroscopy (FCS), which examines time-based changes in the intensity of fluorescently tagged particles moving in Brownian motion, has also been used to study the size of EVs. Recent research by Wyss et al. demonstrated the use of FCS for precise analysis of highly purified EVs from cell culture media by using a built custom algorithm for single-event analysis, allowing for assessment of their concentration based on the expression level of common biomarker CD63 and morphological sizes [159].

A technique for multiplexed biomarker analysis of individual EVs has recently been created, called single EV analysis (SEA), based on fluorescence microscopy including total internal reflection (TIRF-M), confocal, and light-sheet microscopy. In this method, EVs are trapped in a microfluidic device before immunostaining on-chip with up to three common biomarkers and fluorescence images captured. First, the fluorophores already present on the EVs are quenched. Next, three more detection antibodies are added, and the process is repeated for further markers, allowing for the detection of up to 10 different markers on a single vesicle [160,161].

### Surface plasmon resonance (SPR)

SPR biosensor, an optical method, immobilizes a particular receptor protein on active surfaces coated with either gold or silver nanoparticles, enabling susceptible, label-free, and real-time detection of exosomal surface biomarkers. This detection system uses molecular interactions on a surface to quantify the resonant oscillation of electrons generated by incident light at the interface of both a negative and positive dielectric constant material [162]. By focusing on particular biomarkers on the exosome surface, antibodies or oligonucleotides (from 20 to 100 nucleotides) aptamers are stabilized by gold nanoparticles and utilized to quantify tumor-derived EVs [163,164]. This method has been used to evaluate the various forms of functional surfaces, such as gold nanoholes, gold nanopillars, and gold nano-islands, to investigate EVs [[165], [166], [167]]. The last ten years have seen the development of Surface-enhanced Raman Spectroscopy (SERS), a crucial SPR method for the biochemical study of biomarkers with low abundance. The SERS method amplifies the signals, enabling the better analysis of single molecules attached to the composed of gold or silver to metal nanostructures such as iron, nickel, and cobalt with antibody modifications [168,169]. For multiplexed investigation of EVs with low concentrations, an immunolabeled SERS nanoprobe has also been recently created [107,170,171].

### Interferometric reflectance imaging (IRI)

A single particle interferometric reflectance imaging sensor (SP-IRIS) is a digital optical imaging and measuring technique used to analyze individual EVs. This method is based on interferometric imaging of vesicles that affinity agents have captured on a multilayer silicon substrate, where the size is correlated with the contrast of bound vesicles that have been imaged [172]. Sizing and protein profiling of pure vesicles can be conducted in parallel using SP-IRIS in a multiplexed approach. Based on this technique, a platform called "ExoView", which uses cartridges and may detect vesicles as tiny as 40 nm, is currently being developed [162,172,173].

Exosome arrays based on antibodies have recently been developed by NanoView Biosciences.

This technique allows exosome subpopulations to be fractionated using a tiny sample volume. In summary, silicon chips are attached with antibodies against exosome surface biomarkers. Overnight, the chips are treated with exosome solutions or biofluids that include exosomes. Chips are air-dried and then washed with sterile-filtered PBS buffer on a shaker after incubation. SP-IRIS technology detects exosomes by recognizing particles labeled with fluorescence probes in single or multiple color channels. The signal from particles can now have greater contrast due to this method. The sample volume needed for this technology is less and the processing time is shorter than that for flow cytometry.

### Electrochemical sensing (ES)

The electrochemical biosensor is an analytical instrument that identifies molecules such as antibodies and aptamers attached to the exosomal biomarkers. Exosome binding alters the electrochemical signal, which can be utilized to quantify its presence relative to an existing electrical signal. Due to this improved technique's higher sensitivity and speed compared to immunosorbent tests, complex diluted samples can be confidently assessed in the therapeutic application. Electrochemical sensing now enables the examination of samples with concentrations as low as 100 exosomes/ml due to the development of novel capture and detection methods [162,[174], [175], [176], [177], [178]]. In summary, EVs are captured using magnetic beads labeled with specific antibodies to the common exosomal surface biomarkers including CD9, CD63, and CD81 proteins, and the membrane is then ruptured to release the cargo using low-voltage electric cyclic square waves (CSW). The released cargo containing RNAs, and proteins is then hybridized to DNA primers or antibodies on an electrode surface and the change in electrical current is used to measure their concentration. Without chemical lysis, this analysis measures the unusual capacity to measure the cargo of vesicles.

### Western blotting (WB)

SDS-PAGE and immunoblotting are used to determine isolated exosomes in this approach. The Western blotting analysis of exosomes is based on common biomarker receptor proteins (e.g., CD9, CD40, CD63, CD81, and CD151) expressed and localized on the exosomal membrane and internalized proteins such as tumor susceptibility gene 101 protein (Tsg101), Hsp70, Hsp90, and Alix [179]. In this method, proteins are separated through gel electrophoresis, transported to a nitrocellulose membrane, and probed using certain antibodies. Then, immunoreactive bands will be visualized with an imaging system by comparison with known antigen-positive and antigen-negative control samples and molecular weight ladder. However, no specific protein biomarker for exosomes has been established to ensure the isolated sample contains only exosomes. Altogether, a combination of techniques is needed to detect and characterize exosomes, including their purity, quantification, size, and morphology.

## Summary

The significant contribution of exosomes and their content to cell-cell communication is supported by growing research. Exosomes are becoming a popular research topic in pharmaceutical sectors due to their potential use as therapeutic delivery vehicles and non-invasive diagnostic biomarkers. Exosomes have superior properties that strongly support their great potential as early diagnostic biomarkers and therapeutic cargo delivery vehicles in disease treatments, despite the challenges that must still be improved, such as the difficulty of efficient isolation, purification, characterization, and drug loading methods [7,[180], [181], [182]]. The principles, strategies, advantages, and limitations of frequently employed techniques for exosome separation and characterization have been outlined in this review (Table 2). Exosome research is a very new rapidly developing area. The constant development of new methodologies will make translating exosome research to therapeutic applications much easier.

**Table 2:** Comparison of various exosome characterization and detection methods.

Methods	Advantages	Disadvantages	References
Electron microscope (TEM and SEM]	Requires a low amount of samples, High-resolution image, Direct illumination in morphology,	Expensive instrument, Not appropriate for quantitative analysis, Exosomes can be damaged due to dehydration during preparation.	[99,126]
Cryogenic electron microscopy (Cryo-EM)	The absence of the dehydration stage during sample preparation results in no morphological damage to exosomes.	High cost for the instrument, Contamination particles can also be included,	[128,129]
Atomic force microscopy (AFM)	High-resolution and true 3D images with surface topology determinations, Requires a low amount of samples, Staining or fixing is not required.	Expensive instrument, The scanning cantilever can damage exosome morphology, Sample dehydration may cause topographical modifications,	[130,131]
Nanoparticle tracking analysis (NTA)	Straight forward operation, Size variation and concentration can be detected, Available add-on parts for fully automatic operation, Fast detection speed and real-time observation,	Sensitive to vibration, Contamination particles, Inaccurate if samples are aggregated, Expensive instrument,	[[132], [133], [134]]
Field-flow fractionation (AF4)	AF4 is label-free, gentle, fast (<1 h), highly repeatable, and efficient in recovering analytes	Expensive instrument, Users of this technology will require expertise, One inherent restriction is that it divides samples according to their size.	[[139], [140], [141]]
Resistive pulse sensing (RPS)	Higher sampling frequency compared to optical sensing, Applicable in micro/nano-fluidic technology for better sensitivity,	Fabrication with intricate nanostructures, Small sampling efficiency, Calibration is required for every nanopore design,	[142,143]
Tunable resistive pulse sensing (TRPS)	Applicable in characterizing colloidal particles, a wide range of nanoparticles, and biomolecules in suspension,	Hard to select the appropriate nanopore setup,	[144]
Flow cytometry (FCM)	Sub-type EV labeling and detection, Highly sensitive in structural identification and morphological characterization of EVs, Required low sample and is highly repeatable, This method can achieve high-throughput and multi-channel analysis,	Sensitivity limitation in size <200 nm, Laborious and time-consuming, The properties of polydispersity and low refraction limit its application in exosome characterization.	[[147], [148], [149], [150], [151], [152], [153], [154], [155], [156]]
Fluorescence-based analysis (FBA)	Fast and simple, High-resolution, Analysis of a single vesicle,	Expensive instrument and add-on parts, A high sample concentration is required,	[158,161]
Surface plasmon resonance (SPR)	High sensitivity detection in size and quantification of exosomes Integration, miniaturization, multiparameter, real-time, and label-free detection,	Resolution enhancement and detection sensitivity need to be improved.	[[162], [163], [164],^168^]
Interferometric reflectance imaging (IRI)	Sizing and protein profiling of exosomes can be done at the same time, High sensitivity detection in size > 40 nm, Rapid and requires a small volume,	The high degree of background in highly scattered environments, Lower spatial resolution and image quality,	[172,173]
Electrochemical sensing (ES)	Reliable, fast, and easy to handle, Cost-effective, Required low sample concentration, Highly sensitive, Low background,	The sensor applies to a narrow or limited temperature range,	[162,[174], [175], [176], [177]]
Western blotting (WB)	Applicable in the analysis of labeled proteins both qualitatively and quantitatively, Detects a specific target protein by using antibody and compares the expression level with other groups, Applicable in the early detection of disease-related biomarkers,	Complicated and time-consuming, The heating of the buffer can interfere with the transfer, A high concentration of the target proteins is required,	[179]

As a result, to thoroughly analyze the samples, researchers are compelled to use a combination of procedures depending on their hypothesis. However, most of the recent EV analysis methods demand expensive equipment and expertise that might not be widely available. Technologies that can enhance exosome isolation and characterization are developing, and they are also expected to help improve our knowledge of the function of tumor-derived exosomes in the growth and metastasis of cancer, as well as the creation of effective anticancer therapy.

### Future directions for EV isolation and characterization

One of the difficulties in generating, separating, and employing exosomes for therapeutic applications is preserving exosomes so that they may be transported, stored, and applied without causing their cargo damage for an extended time. Current EV isolation and characterization methods are expected to advance, opening novel opportunities for in-depth study of EV biogenesis and expanding our understanding of their structure and functional characteristics. Every technique selects a specific isolation and characterization method with advantages and disadvantages. In addition, it is crucial to consider enhancing the detection limits and resolution when considering the optimization of novel approaches. This field must spend more effort to develop next-generation processes that would be a gold standard as faster, more sensitive, require fewer steps, cost-effective, and reliable for the efficient separation of exosomes. Meanwhile, plant-derived exosome-like nanoparticles (PELNs) are currently being investigated for their potential use as an alternative to animal cell-derived exosomes (ADEs), allowing researchers to avoid the technical restrictions of ADEs [[183], [184], [185]]. However, the safety, stability, and biocompatibility of PELNs pathways in animal models should be thoroughly investigated. I believe that technological improvements in isolation, characterization, sensitivity, and drug delivery approaches of exosomes increase their efficacy in different therapeutic applications.

## Conclusions

This review covers recent conventional methods used in laboratories worldwide to separate, detect, and characterize exosomes. Low yields and purity, high costs, overly complicated methods, and a lack of standardization are issues that the field must develop for research and the practical use of exosomes in clinical trials. Due to novel techniques, detecting exosomes in small clinical samples is now possible. Since EVs are present in human fluids, particularly blood, saliva, and urine, EV-based liquid biopsy holds incredible promise for personalized therapy. For instance, developing electrochemical biosensor devices, such as iMEX, that can measure exosomes from blood samples is encouraging since it opens the door to liquid biopsy as a point-of-care tool for diagnosis and therapy response prognosis of cancer.

However, all the models and methodologies presented in this field will still require considerable preclinical experience to verify reliability. To understand the function that exosomes play in the development of diseases, the molecular contents of exosomes must be more thoroughly defined in both health and disease. One of the current challenges with such investigations is identifying distinctive biological activities based on EV subtypes. Although some of these functional distinctions have been attributed to certain sEV and lEV populations, this knowledge will require a more comprehensive definition of EV subtypes based on their biogenesis mechanism or cargo content. Additionally, different isolation and purification methods may be utilized depending on the purpose of the exosomes, such as whether they are being used for diagnosis or therapy. Undoubtedly, brand-new methodologies will continuously grow and improve because of the broad interest in exosomes from academics and the pharmaceutical sectors which will aid in exosome research in clinical applications shortly. Finally, when selecting, combining, and optimizing exosome isolation methods for clinical applications, the type of sample, sample quantity, equipment availability, processing time, labor cost, therapeutic purposes, administration route, and desired final product should all be considered.

## Informed consent statement

Not applicable.

## Data availability statement

Not applicable.

## Funding

This research does not receive any external funds.

## Ethics approval and consent to participate

Not applicable.

## Consent for publication

Yes.

## CRediT authorship contribution statement

**Nihat Dilsiz:** Writing – review & editing, Writing – original draft, Supervision, Methodology, Investigation, Data curation, Conceptualization.

## Declaration of competing interest

I am not interested in conflicts. I have not received funding from any agency.
